# The Role of Serum and Peritoneal Biomarkers in Predicting Sepsis and Septic Multiorgan Failure in Patients With Secondary Peritonitis

**DOI:** 10.7759/cureus.41724

**Published:** 2023-07-11

**Authors:** Clementina O Dumitrascu, Mihai Gherghe, Mihai Costache, Bogdan Cretu, Catalin Cirstoiu

**Affiliations:** 1 Anesthesiology and Critical Care Department, University Emergency Hospital of Bucharest, Bucharest, ROU; 2 Orthopedics and Traumatology Department, University Emergency Hospital of Bucharest, Bucharest, ROU

**Keywords:** syndrome, cytokine storm, peritoneal cytokines, sepsis, secondary peritonitis

## Abstract

Purpose

Secondary peritonitis is still one of the most important causes of severe sepsis in the world; therefore, it is of utmost importance to identify biomarkers that could be employed for the purpose of selecting patients at high risk for developing life-threatening complications after emergency surgery. In view of this quest, our study seeks to reveal the possible role for serum and peritoneal concentrations of selected biomarkers, specifically presepsin, procalcitonin, monocyte chemoattractant protein-1 (MCP-1), high mobility group box 1 protein (HMGB-1) and interleukins (IL-6, -8, -10), in early prediction of sepsis and septic multiorgan failure for patients with secondary peritonitis.

Methods

We prospectively observed 32 selected patients with secondary peritonitis that underwent emergency surgery. Blood and peritoneal fluid samples were drawn at the time of surgery (T0), and after that, blood samples were taken at 24 (T1) and 48 (T2) hours postoperatively. Cytokines concentrations were determined using a sandwich enzyme-linked immunosorbent assay (ELISA), a non-competitive variant, both in peritoneal fluid and serum.

For determining whole blood concentration of presepsin and procalcitonin, PATHFAST™ assays (Polymedco, Cortlandt, New York) were used, based on the principle of non-competitive chemiluminescent enzyme immune-assay (CLEIA).

The study was conducted according to the guidelines of the Declaration of Helsinki and approved by the Ethics Committee of University Emergency Hospital Bucharest (no. 40325/6 April 2023).

Results

We found significant elevations in the peritoneal concentrations of interleukins 6, 8, 10, HMGB-1, and MCP-1 in all patients with secondary peritonitis at the moment of surgery; however, no clear correlation could be made based on this data with patient evolution. With regards to blood concentrations of the aforementioned serum cytokines and presepsin, procalcitonin (as already established markers of sepsis), our results showed good predictive value of presepsin for developing sepsis and septic multiorgan failure from the first hours in this patient category. All other biomarkers, despite having higher concentrations than baseline, in particular at 24-48 hours after surgery, had unpredictable dynamics that couldn’t be correlated with the severity of the disease.

Conclusion

Cytokine production is the mainstay in developing sepsis and septic multiorgan failure in patients with secondary peritonitis; therefore, studying the dynamics of said cytokines seems of interest in finding tools to predict the development of sepsis or sepsis-related mortality. However, at the time, there seemed to be no clear correlation between the values of these cytokines and the development of complications.

## Introduction

Secondary peritonitis represents the inflammation of the peritoneum, caused typically by the spread of bacteria and contents of intraabdominal organs into the peritoneal cavity. The initiating factor is most frequently a functional or physical breaching of the integrity of the gastrointestinal tract, produced either through perforation (direct contamination) or due to other pathological processes (intestinal ischemia, volvulus, intraperitoneal hemorrhage secondary to trauma [1]), associated with intraabdominal hypertension. Secondary peritonitis remains an important pathology in critical care patients worldwide due to the possible severe consequences of developing intraabdominal manifested sepsis, with high rates of mortality. Improving outcomes and reducing mortality through early diagnosis, prognostication, and treatment of infection [2] still represents a major challenge for the clinician due to the rapid and unpredictable, dysregulated host response to infection in this type of patient.

Sepsis syndrome, as a result of interactions between the host and insults generated by the infectious process, leading to the activation of pro-inflammatory and anti-inflammatory responses through the production and release of biochemical mediators, remains a life-threatening illness. Damage-associated molecular patterns (DAMPs) released from injured tissues, and the appearance of pathogen-associated molecular patterns (PAMPs) produced in bacterial infections, activates pattern recognition receptors (PRRs), including endothelial and immune cell Toll-like receptors (TLRs). This process promotes the activation of different pathways, which conduct the production of multiple inflammatory mediators (interleukins 6, 8, 1/β, 10, monocyte chemoattractant protein-1 (MCP-1), tumor necrosis factor TNF-α, thromboxane A2, high mobility group box 1 protein (HMGB-1), thrombin and others) acting as effectors and triggering a systemic inflammatory response, sepsis, coagulopathy, tissue edema and hypoxia, finally cellular dysfunction and multiorgan failure [2]. 

The intra-abdominal contamination that occurs in patients with secondary peritonitis represents a large reservoir of PAMPs and DAMPs and triggers an extensive systemic response with cytokine discharge [1].

Procalcitonin (PCT) is a pro-peptide of calcitonin hormone, which rapidly rises in plasma, stimulated by bacterial endotoxins. Assicot et al. first described significantly increased concentrations of PCT in patients with bacterial and fungal infections and sepsis [3]. Since then, it has been confirmed in many studies that PCT closely correlates with the inflammatory host response to microbial infections [4-6]. The assay technique is today easy to use, and plasma PCT is evaluated in septic patients from admission to the emergency department.

Presepsin (sCD14-ST) is a lipopolysaccharide (LPS) receptor on myeloid cells, a member of the family of Toll-like receptors. Soluble CD14 fragments (presepsin) are cleaved in inflammation and can be ease measured, rising rapidly in the first two hours of either localized or systemic infection, with rather good sensitivity and specificity. Because presepsin plays an important role in sepsis and is relatively specific for bacterial infection, it became a useful biomarker for diagnosis, prognostic and risk stratification in septic patients [7-8].

This study seeks to reveal the possible role of selected serum and peritoneal biomarkers concentrations, specifically presepsin, procalcitonin, MCP1, HMGB-1, and interleukins 6, 8, 10, for a more accurate prediction of sepsis and septic multiorgan failure in patients with secondary peritonitis.

## Materials and methods

Study design and settings 

This prospective observational study was conducted at the department of critical care medicine of The University Emergency Hospital of Bucharest over a period of three years. The lot of study included 32 patients with secondary peritonitis who had undergone emergency laparotomy.

The study was conducted according to the guidelines of the Declaration of Helsinki and approved by the Ethics Committee of University Emergency Hospital Bucharest (no. 40325/6 April 2023).

Study population

The population included 32 patients with secondary peritonitis who met inclusion criteria, meaning they were 18 years old with acute peritonitis, had agreed to participate in the study, and did not have any exclusion criteria such as having undergone recent chemotherapy, peritoneal dialysis that was under treatment with immunosuppressive agents, or had preexisting ascites.

Data collection

Blood and peritoneal fluid samples were taken at the time of the surgery (T0); samples were centrifuged at 1300 g for 10 min, within 30 minutes of collection. The serum obtained was stored initially at -20 °C in the ICU department and then transferred to the molecular pathology laboratory in our hospital, for -80 degrees °C storage, until sample processing. Blood sampling was repeated at 24 (T1) and at 48 (T2) hours after surgery.

Other routine laboratory test results and clinical data from the patients were collected daily, and the treatments applied have been in accordance with standard guidelines. 

The measurements of plasma and peritoneal fluid levels of interleukins 6, 8, 10, and MCP-1, HMGB-1 were made by the same investigator by complying with the technique of sandwich enzyme-linked immunosorbent assay (ELISA) non-competitive variant.

Data management and statistical analysis

Data was collected, stored, and formatted using a combination of Office 360 tools (Microsoft, Redmond, Washington) and iOS Pages (Apple Inc., Cupertino, California). The median, mean values, and graphs were further done and edited in Excel.

Statistical analysis was not performed due to the small sample of the study population and lack of homogeneity in the causes of secondary peritonitis leading to an underpowered study.

Purpose

This study seeks to reveal the possible role of selected serum and peritoneal biomarkers concentrations, specifically presepsin, procalcitonin, MCP1, HMGB-1, and interleukins 6, 8, 10, for a more accurate prediction of sepsis and septic multiorgan failure in patients with secondary peritonitis.

## Results

Thirty-two patients were selected for the study and assigned into three groups: non-septic, septic, and sepsis-induced multiple organ dysfunction syndrome (MODS). Patients were assigned to a septic vs. non-septic group based on the Sepsis-3 criteria of sepsis. All patients were screened by quick Sepsis Related Organ Failure Assessment (qSOFA) at admission. Patients in the sepsis-induced MODS group were all septic patients which further developed multiple organ system failure and were as such distributed to the group considering the difference in mortality between the two groups. All patients underwent emergency surgery for secondary peritonitis. Most patients were male, but in the septic and sepsis induced MODS group were older (median age 67 and 70) than the non-septic group (median age 40). The distribution of the causes of secondary peritonitis is shown in Table 1. The main event leading to secondary peritonitis was perforation of cavity organs. Other causes were intestinal obstruction, ruptured abscesses or anastomotic leakage. Two patients in the sepsis induced MODS group died by T1 (24h after surgery) and another two by T2 (48h after surgery).

**Table 1:** The distribution of the causes of secondary peritonitis MODS - multiple organ dysfunction syndrome

Characteristic	Non-septic	Septic	Sepsis-induced MODS
Total No.	10/32	7/32	15/32
Age, median (y)	40	67	70
Male, No/total (%)	6/32 (18%)	5/32 (15%)	8/32 (25%)
Type of admission, No./total
Perforation	7/32	5/32	7/32
Acute intestinal obstruction	0/32	2/32	6/32
Anastomotic leakage	0/32	0/32	1/32
Ruptured abscess	3/32	0/32	1/32
Renal dysfunction, No./total cat.	1/10	2/7	13/15

At T0, all patients had full blood count, coagulation panel, fibrinogen concentration, comprehensive metabolic panel, presepsin, and semi-quantitatively procalcitonin.

**Figure 1:**
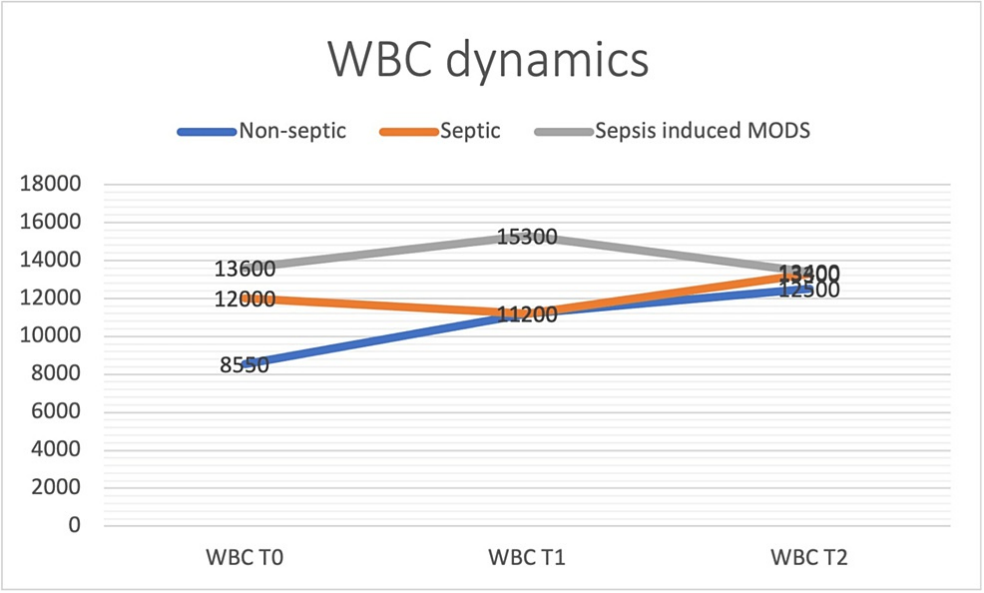
Patients with sepsis and sepsis-induced MODS had higher WBC count at T0, with peak levels at T1 for patients with sepsis-induced MODS and peak levels at T2 for patients in the sepsis group MODS - multiple organ dysfunction syndrome

Patients with sepsis and sepsis-induced MODS had higher WBC count at T0, with peak levels at T1 for patients with sepsis-induced MODS and peak levels at T2 for patients in the sepsis group (Figure 1).

**Figure 2:**
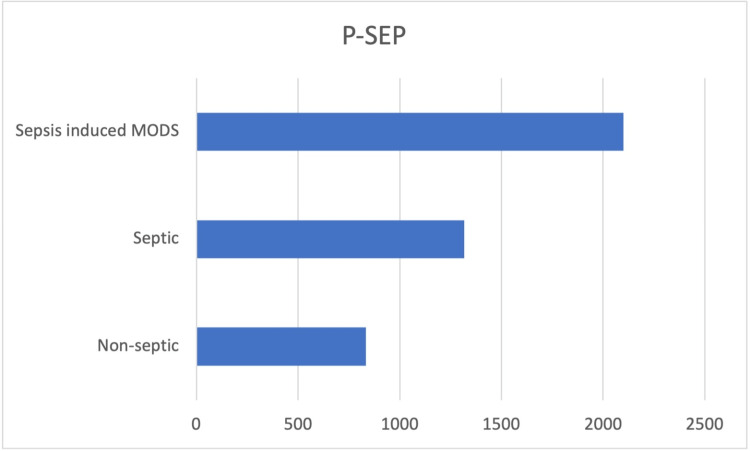
Presepsin levels were higher in the sepsis and sepsis-induced MODS groups (median value 1316 pg/ml and 2100 pg/ml, respectively) MODS - multiple organ dysfunction syndrome

Presepsin levels were higher in the sepsis and sepsis-induced MODS groups count at T0 (median value 1316 pg/ml and 2100 pg/ml, respectively) (Figure 2).

**Figure 3:**
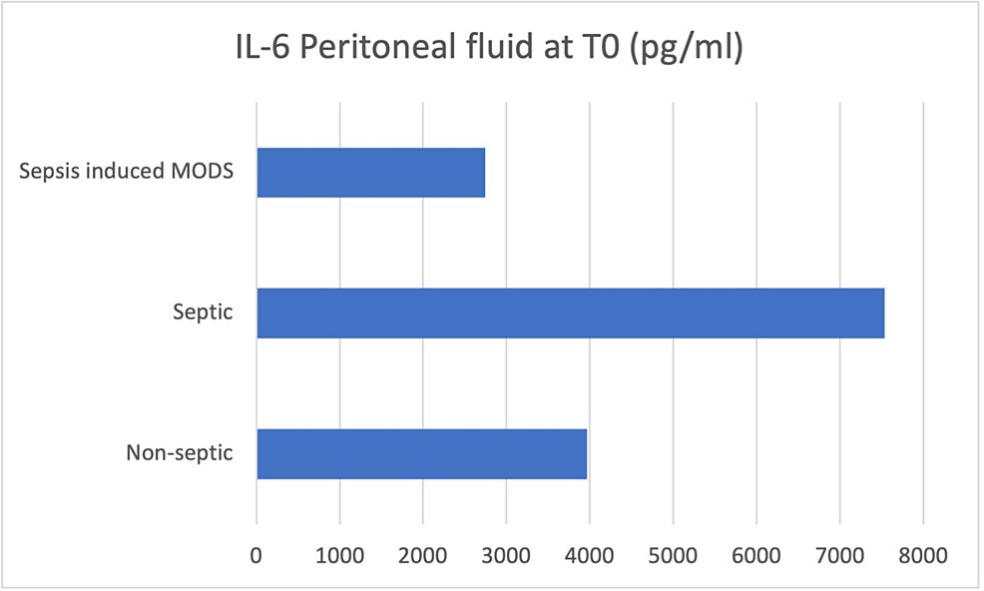
Peritoneal fluid samples were taken intraoperatively (T0), and the concentrations of IL-6 was determined

**Figure 4:**
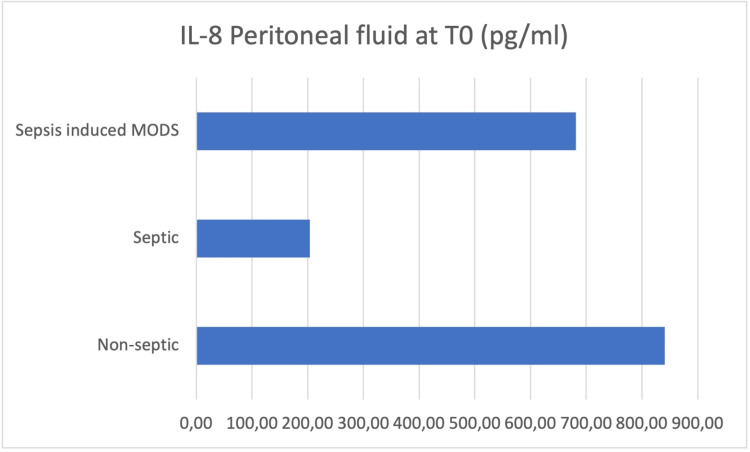
Peritoneal fluid samples were taken intraoperatively (T0), and the concentrations of IL-8 were determined

**Figure 5:**
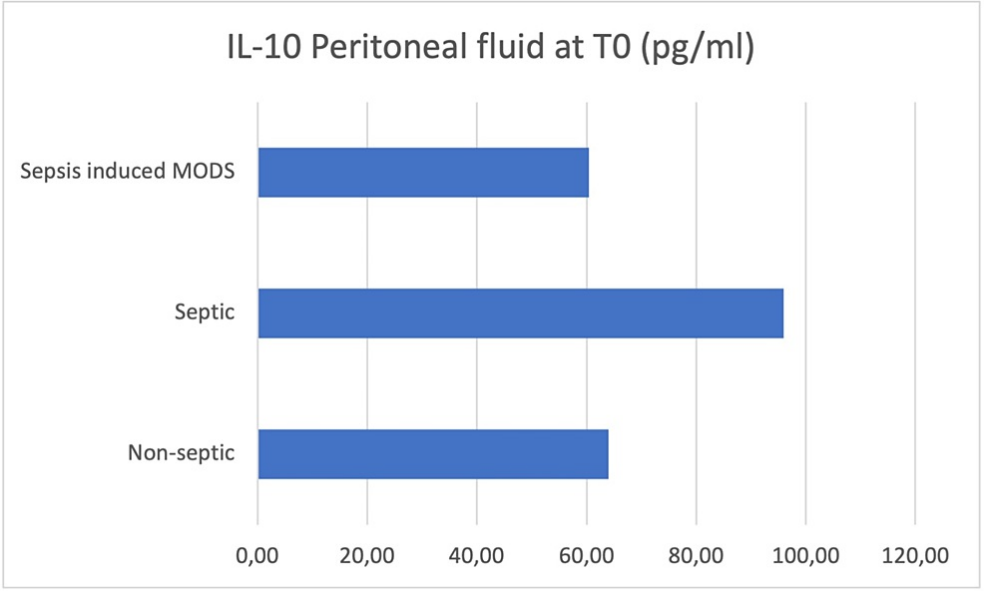
Peritoneal fluid samples were taken intraoperatively (T0), and the concentrations of IL-10 were determined

**Figure 6:**
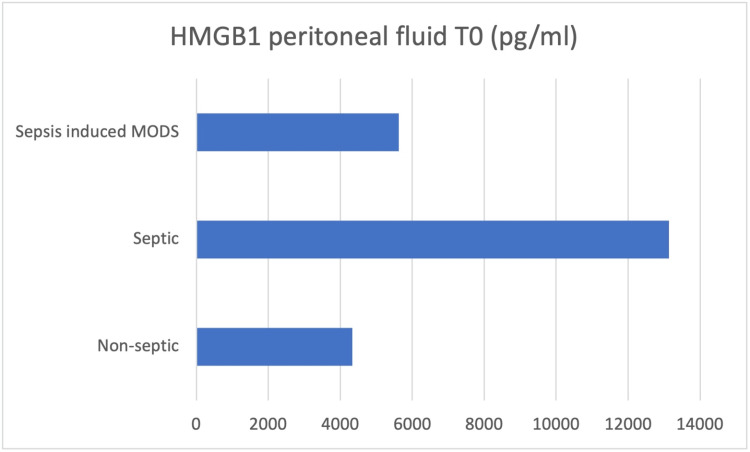
Peritoneal fluid samples were taken intraoperatively (T0), and the concentrations of HMGB-1 were determined HMGB-1 - high mobility group box 1 protein

**Figure 7:**
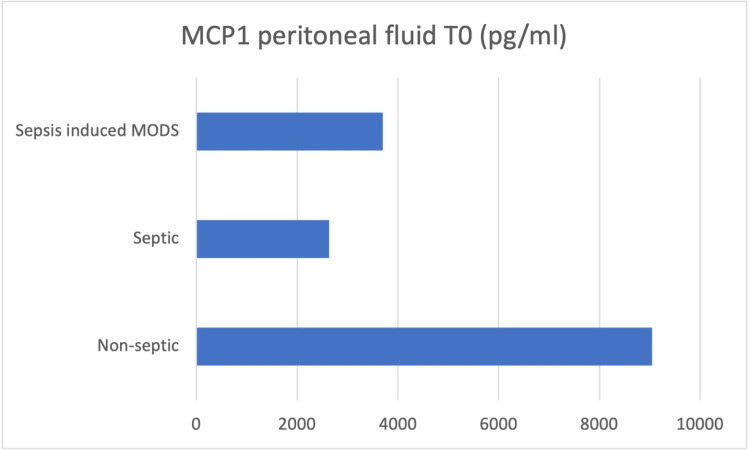
Peritoneal fluid samples were taken intraoperatively (T0), and the concentrations of MCP-1 were determined MCP-1 - monocyte chemoattractant protein-1

Peritoneal fluid samples were taken intraoperatively (T0), and the concentrations of IL-6, IL-8, IL-10, HMGB-1, and MCP-1 were determined (Figures 3-7). 

At the moment of surgery (T0), the results showed concentration values of IL-6, IL-10, and HMGB-1 higher in the peritoneal fluid of septic patients, while IL-8 and MCP-1 concentrations were higher in the peritoneal fluid of non-septic group of patients.

**Figure 8:**
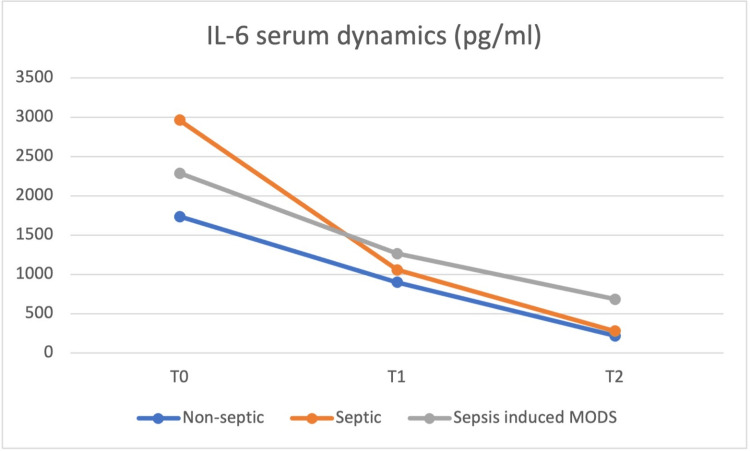
IL-6 plasma concentrations at T0 were highest in the septic group, but from T1 until T2, the sepsis-induced MODS group of patients presented the highest concentrations MODS - multiple organ dysfunction syndrome

**Figure 9:**
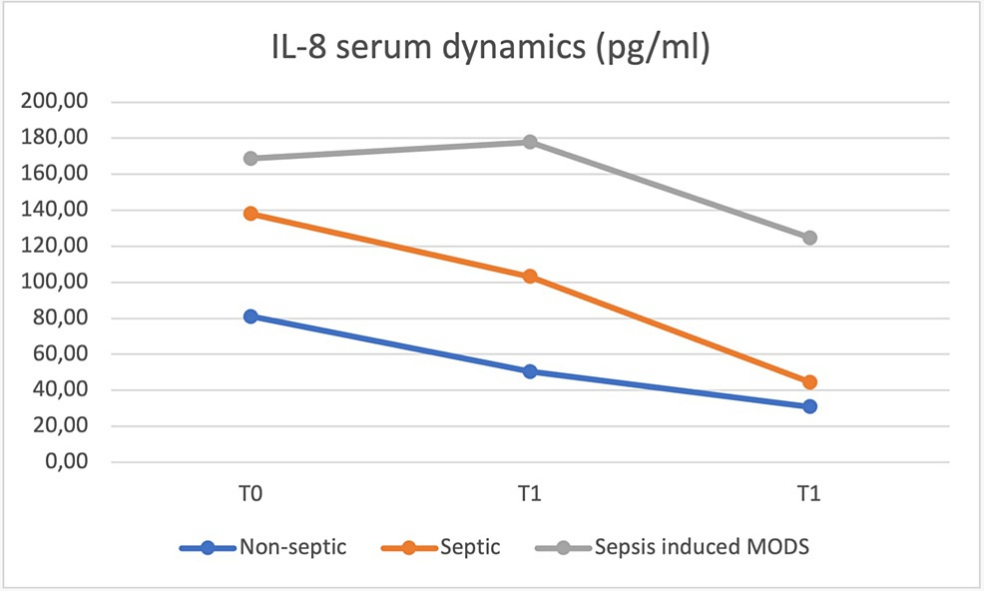
IL-8 concentrations were highest in the sepsis-induced MODS group at all moments, from T0 to T2 MODS - multiple organ dysfunction syndrome

**Figure 10:**
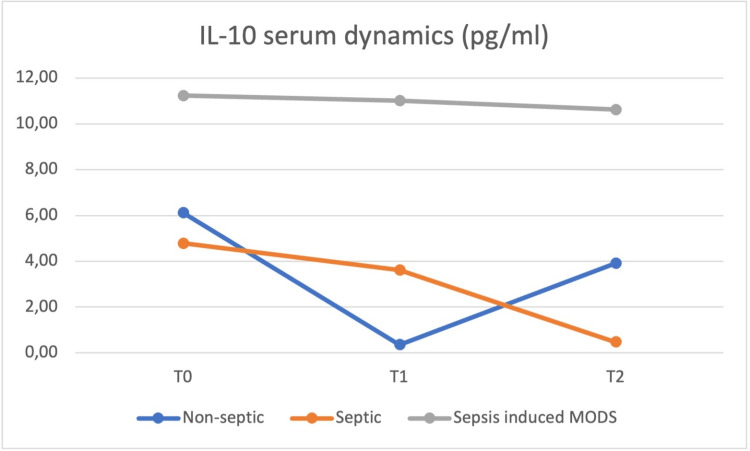
IL-10 concentrations was highest in the sepsis-induced MODS group at all moments, from T0 to T2 MODS - multiple organ dysfunction syndrome

**Figure 11:**
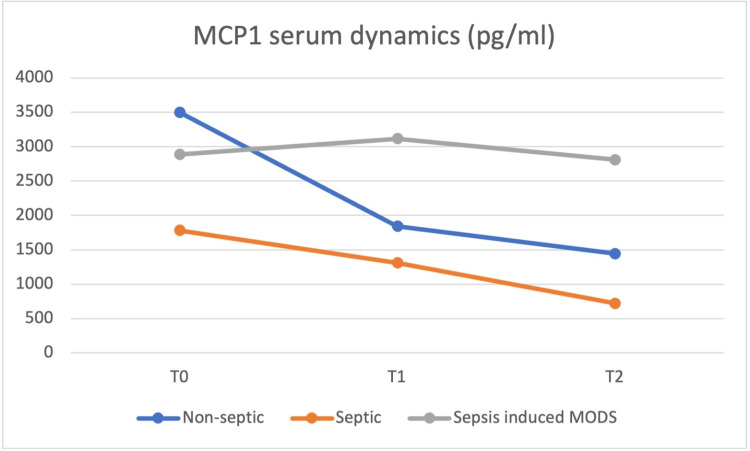
MCP-1 plasma concentrations at T0 were highest in the non-septic group, but from T1 until T2 the sepsis-induced MODS group of patients presented the highest concentrations MCP-1 - monocyte chemoattractant protein-1, MODS - multiple organ dysfunction syndrome

**Figure 12:**
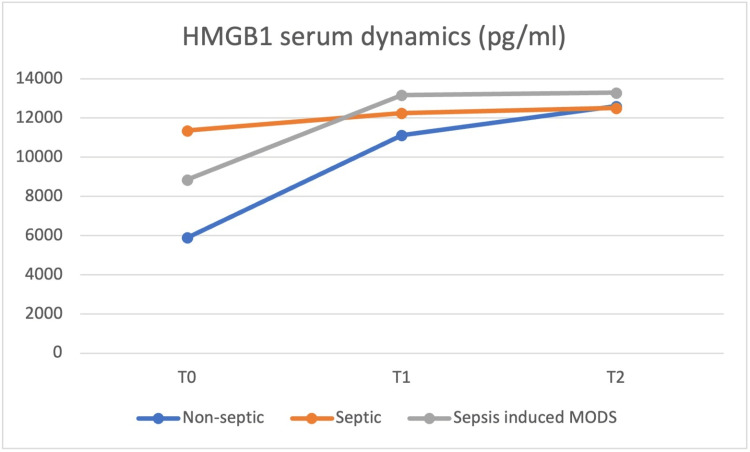
HMGB-1 plasma concentrations at T0 were highest in the septic group, but from T1 until T2, the sepsis-induced MODS group of patients presented the highest concentrations HMGB-1 - high mobility group box 1 protein, MODS - MODS - multiple organ dysfunction syndrome

At the same time (T0), blood samples were obtained, and the values of the same cytokines were determined and repeated at 24h (T1) and at 48h (T2) postoperatively, with the dynamics of the serum values presented in Figures 8 to 12. 

IL-8 and IL-10 concentrations were highest in the sepsis-induced MODS group at all moments, from T0 to T2. IL-6 and HMGB1 plasma concentrations at T0 were highest in the septic group, but from T1 until T2, the sepsis-induced MODS group of patients presented the highest concentrations. HMGB-1 showed a relatively constant concentration in the plasma of septic patients, with a T0 level above that of non-septic and sepsis-induced MODS patients, decreasing at T1 below the levels of the sepsis-induced MODS group of patients and maintaining similar levels at T2.

Based on the data obtained in our study, no clear correlation could be made between these values and patient severity, but a similar pattern for the sepsis-induced MODS group of patients was observed, with the highest plasmatic concentrations for all biomarkers from T1 to T2.

## Discussion

Even though the population in this prospective observational study included only 32 patients, we can observe that presepsin is useful in the stratification of patients at risk of developing sepsis and septic multiorgan failure after secondary peritonitis. We evaluated presepsin levels at the time of surgery and obtained the highest values in patients that developed MODS. Our findings were similar to multiple recent studies assessing the ability of presepsin to predict septic shock and MODS. 

The values of the biomarkers determined in the peritoneal fluid samples at the time of surgery, intriguing enough, did not correlate with the development of sepsis or septic multiorgan failure. The highest concentrations of these cytokines were found in the septic group and, therefore, could not be used to predict organ dysfunction, as these patients had lower values of said cytokines across the range. Our findings are contradictory to some other recent publications that show a trend toward mortality and adverse outcomes in patients with a more reactive peritoneal cavity.

Lee et al., in 2022, suggested a high sensitivity of presepsin in predicting sepsis and septic multiorgan failure at values above 821 pg/ml in a prospective observational study conducted on 420 patients [9].

The concentration of IL-6 and HMGB-1 in plasma was highest in the septic group at T0, while IL-8 and IL-10 had the highest serum concentrations in the MODS group. A group of studies claim somewhat of an anti-inflammatory role of IL-10 [10-14], which did not result in our own findings. The behavior of plasmatic IL-10 was characterized in other studies more as a potent immunosuppressive cytokine which is increased in the plasma of patients with septic shock and associated with increased mortality and a higher rate of nosocomial infections [15-18], this behavior being more similar to our study. 

However, some studies point to the possibility that higher values of IL-8 and MCP-1 in plasma can also predict the development of organ failure and mortality, findings similar to our own between 24 to 48 hours postoperatively. 

The need to find better tools for clinicians in their day-to-day practice is an ongoing preoccupation for the medical world. Targeting a combination of peritoneal and serum biomarkers to early identify the patients at risk of developing life-threatening complications after emergency surgery for secondary peritonitis is a point of interest for future studies on a larger number of patients. Future research should focus on establishing cutoff values for selected and/or combined inflammatory cytokines in the peritoneal fluid and serum at different moments to better stratify the patients at risk of developing severe septic complications.

Patients that had undergone recent chemotherapy, peritoneal dialysis that was under treatment with immunosuppressive agents, or had preexisting ascites were excluded from the study. 

## Conclusions

Cytokine production is the mainstay in developing sepsis and septic multiorgan failure in patients with secondary peritonitis; therefore, we proposed measurements in plasma and peritoneal fluid, at different moments in time, of interleukins (IL-6, IL-8, IL-10), and MCP-1, HMGB-1 concentrations, together with presepsin and procalcitonin in plasma, for determining their value in stratifying the risk of developing sepsis and septic multiorgan dysfunction in this category of patients. 

We concluded that out of all the biomarkers studied, presepsin was most likely to predict sepsis and septic multiorgan failure from the first hours, while the rest of the biomarkers determined, despite the rising of their plasmatic levels at 24-48 hours after surgery, had unpredictable values, thus requiring more studies on larger populations.
